# Classical risk factors of cardiovascular disease among Chinese male steel workers: a prospective cohort study for 20 years

**DOI:** 10.1186/1471-2458-11-497

**Published:** 2011-06-25

**Authors:** Jingfeng Ji, Enchun Pan, Jianxin Li, Jichun Chen, Jie Cao, Dongling Sun, Xiangfeng Lu, Shufeng Chen, Dongfeng Gu, Xiufang Duan, Xigui Wu, Jianfeng Huang

**Affiliations:** 1 Department of Evidence Based Medicine, Cardiovascular Institute and Fu Wai Hospital, Chinese Academy of Medical Sciences and Peking Union Medical College, Beijing, China; 2 Cardiovascular Institute and Fu Wai Hospital, Chinese Academy of Medical Sciences and Peking Union Medical College, Beijing, China

## Abstract

**Background:**

Cardiovascular disease (CVD) constitutes a major public health problem in China and worldwide. We aimed to examine classical risk factors and their magnitudes for CVD in a Chinese cohort with over 20 years follow-up.

**Methods:**

A cohort of 5092 male steelworkers recruited from 1974 to 1980 in Beijing of China was followed up for an average of 20.84 years. Cox proportional-hazards regression model were used to evaluate the risk of developing a first CVD event in the study participants who were free of CVD at the baseline.

**Results:**

The multivariable-adjusted hazard ratio (HR) associated with every 20 mmHg rise in systolic blood pressure (SBP) was 1.63 in this Chinese male population, which was higher than in Caucasians. Compared to non-smokers, men who smoked not less than one-pack-a-day had a HR of 2.43 (95% confidence interval [CI], 1.75-3.38). The HR (95% CI) for every 20 mg/dl increase in total serum cholesterol (TC) and for every point rise in body mass index (BMI) was 1.13 (1.04-1.23) and 1.06 (1.02-1.09), respectively.

**Conclusions:**

Our study documents that hypertension, smoking, overweight and hypercholesterolemia are major conventional risk factors of CVD in Chinese male adults. Continued strengthening programs for prevention and intervention on these risk factors are needed to reduce the incidence of CVD in China.

## Background

Cardiovascular disease (CVD) constitutes a major public health problem in China [1] and worldwide [2]. Classical risk factors for coronary heart disease (CHD) and stroke have been identified [3,4], such as high blood pressure, tobacco smoking, hypercholesterolemia, obesity and age. The roles of major cardiovascular risk factors in the development of CHD or stroke are well established in Western populations. While for Chinese, data are less extensive, especially lack of over twenty years prospective cohort study to investigate the magnitude of classical risk factors for CVD. Previous studies have proved that the profile of CVD among Chinese is much different from among Caucasians. Compared with Western populations, the incidence and mortality rates of cerebrovascular disease were much higher than those of CHD in Chinese populations [5]. Risk factors of developing acute coronary syndrome, ischemic or hemorrhagic stroke were explored in a multi-provincial cohort with 10 years follow-up [6], and results of classical risk factors for CHD and stroke in our cohort with 13.5 years follow-up were reported previously [7,8], however, it is unclear the profile and magnitude of classical risk factors for CVD in a Chinese cohort with over 20 years follow-up, and whether it was different from that in Western populations. Therefore, we aimed to examine classical risk factors and their magnitudes for CVD in this Chinese cohort.

## Methods

### Study population

A prospective cohort study of CVD has been conducted in the Beijing Iron and Steel Complex since 1974. Details of the study population and methods for the cohort have been published elsewhere [7]. In 3 separate waves of recruitment (1974, 1979, and 1980), 5298 male workers(aged 18 to 74 years) from 7 factories of the Beijing Iron and Steel Complex attended health check, and 5092 participants were free of CVD at the baseline. The project health practitioners conducted follow-up until 2001. By the year of 2001, 106 participants were lost to follow-up and 4986 participants were included in our cohort.

### Baseline examination

In the 1974, 1979, and 1980 surveys, clinical evaluation and laboratory measurements were performed with the same protocol. Demographic lifestyle information and measurements including blood pressure, total cholesterol (TC), weight, and height were obtained. Smoking and drinking behaviors were collected by the interview. Blood pressure was measured in the right arm using a standard mercury sphygmomanometer with the subject in a sitting position. Hypertension was defined as systolic blood pressure (SBP) more than or equal to 140 mmHg and/or diastolic blood pressure (DBP) more than or equal to 90 mmHg. Pulse pressure (PP) was defined as PP = SBP-DBP, and mean arterial pressure (MAP) was calculated from the standard equation MAP = (2/3) DBP+ (1/3) SBP (in mmHg). The height and weight of each subject, wearing light clothes without shoes, were recorded. Body mass index (BMI) was calculated as weight (kg)/height (m)^2^. TC was determined on the same single occasion from a fasting venous blood sample, using the enzyme regent method as our previous study reported [7]. Diabetes was not included in our analyses because only six persons reported diabetes at baseline.

### Follow-up data collection

We carried out follow-up survey in 1982, 1987, 1993 and 2001, respectively. By the year of 2001, 2.1% of subjects were lost to follow-up. The average follow-up time was 20.84 years. If participants could not be followed up by a way of face-to-face or telephone interview, their relatives or colleagues were contacted for information. Because surveillance system of CVD has been set up since 1960s in Beijing Capital Steel and Iron Company and most of steelworkers were treated in Capital Steel Hospital, self-reported outcomes could be confirmed by reviewing disease report cards and hospital records. When participants reported CVD, their hospital records were verified by physicians from the company hospital, and by physicians from the cardiovascular institute and Fu Wai hospital, Chinese Academy of Medical Sciences. For all fatalities, death certificates were obtained to confirm cause of death.

This study defined CVD as a composite of CHD events (including acute myocardial infarction [AMI], coronary sudden death and other coronary death) and cerebrovascular events (including ischemic stroke and hemorrhagic stroke), but not peripheral vascular disease. Coronary deaths included deaths caused by AMI, heart failure and cardiac arrest.

This research was carried out in compliance with the Helsinki Declaration. Ethics committees in Cardiovascular Institute and Fuwai Hospital, Chinese Academy of Medical Sciences approved the study with a reference number 110-1.

### Statistical Analyses

This analysis was performed with the 4238 individuals who had no missing values for the variables under investigation. We compared the baseline characteristics of the remaining population with that of the original population. Cox proportional-hazards regression model was used to estimate the hazard ratios (HRs) of CVD incidence for preventable risk factors. Covariates included in Cox models were age, hypertension, cigarette smoking, TC, and BMI. Participants who had both CHD and stroke event were censored after their first CVD event in Cox model. Considering the problem of collinearity, blood pressure indices (SBP, DBP, MAP and PP) were separately included in the multivariate regression model with other risk factors. The cut points of adult underweight, overweight and obesity according to BMI were defined by the Chinese overweight and obesity prevention guideline [9] and the World Health Organization (WHO) classification [10], respectively. We also tested for interactions between each variable and age, and compared our results with the results of version two of the QRISK cardiovascular disease risk algorithm (QRISK2) [11].

The population-attributable risk (PAR) was also calculated to quantify the contribution of each independent modifiable risk factor on CVD using the following equation: PAR = [P × (RR-1)]/[P × (RR-1)+1], where P signifies the prevalence of the risk factor and RR signifies the adjusted relative risk of CVD incidence [12]. Because the risk factor levels might change a lot during the last two decades in China, it wasn't very convincible to use the prevalence rates of these risk factors at the baseline. Therefore we used the prevalence in our study and the prevalence for urban male adults (18 years of age or older) in the National Health and Nutrition Survey 2002 [13,14] to calculate the PAR. The HR from Cox regression models was used to estimate relative risk (RR). The statistical package SPSS (version 12.0, Chicago, USA) was used for data analysis.

## Results

### Baseline Characteristics of the Study Population

The baseline characteristics of the original population and the remaining population in this analysis are presented in Table 1. The remaining population was representative of the original population. During the follow-up, 391 subjects developed a first CVD event in the remaining population, including 255 cases of stroke and 136 cases of CHD events. A total of 599 deaths were reported, 240 persons died of CVD, 194 persons died of neoplasm and 165 persons died of other disease and injury.

**Table 1:** Baseline characteristics of the Beijing steel workers cohort

Variables	Entire cohort (N = 4986)	means ± SD	Cohort used in this study (N = 4238)	means ± SD
Age (yr)	4975	45.08 ± 7.82	4238	44.91 ± 7.93

TC (mg/dl)	4521	186.25 ± 38.65	4238	187.58 ± 37.87

BMI (kg/m^2^)	4782	23.18 ± 2.72	4238	23.17 ± 2.71

SBP (mmHg)	4976	123.26 ± 18.86	4238	122.89 ± 18.67

DBP (mmHg)	4976	80.91 ± 11.89	4238	80.80 ± 11.83

PP (mmHg)	4976	42.34 ± 11.78	4238	42.09 ± 11.77

MAP (mmHg)	4976	95.03 ± 13.49	4238	94.83 ± 13.37

Hypertension (%)	4976	32.13	4238	31.69

Cigarette smoker (%)	4932	73.44	4238	73.36

### Multivariate analyses of classical risk factors

Table 2 shows the results of the Cox regression analyses for the final model. There were continuous, graded increases in CVD risk either for every 20 mmHg rise of SBP, or for every 10 mmHg rise of DBP. For the men with the highest level of blood pressure(SBP ≥ 180 mmHg or DBP ≥ 100 mmHg) had the highest risk of CVD, and the HRs (95% confidence interval [CI]) was 7.56(4.71-12.14) and 4.19(3.07-5.71), respectively. Compared to non-smokers, the multivariable adjusted HR of CVD was 2.43 (95% CI, 1.75-3.38) for men who smoke more than or equal to one-pack-a-day. In multivariate analysis, there was an increased risk of CVD only in the group of TC category greater than or equal to 220 mg/dL, with a HR of 1.42(95%CI, 1.08-1.85).

**Table 2:** Multivariable adjusted hazard ratios for CVD according to different levels of major risk factors

Variables	Range	CVD events	Number	Person-years	Incidence (per 1000 person years)	HR	95%CI
SBP (mmHg)	< 120	88	1712	35895	2.45	1	-

	120-139	136	1641	33646	4.04	1.52	1.16-1.99

	140-159	99	635	12451	7.95	2.48	1.84-3.36

	160-179	45	194	3465	12.99	3.59	2.47-5.24

	≥ 180	23	56	831	27.68	7.56	4.71-12.14

DBP (mmHg)	< 80	75	1529	31955	2.35	1	-

	80-89	111	1500	30971	3.58	1.49	1.11-2.00

	90-99	96	751	15015	6.39	2.27	1.67-3.10

	≥ 100	109	458	8347	13.06	4.19	3.07-5.71

PP (mmHg)	< 35	72	1099	23008	3.13	1	-

	35-44	136	1737	35663	3.81	1.15	0.87-1.54

	45-54	93	909	18289	5.09	1.41	1.03-1.93

	≥ 55	90	493	9328	9.65	2.08	1.50-2.87

MAP (mmHg)	< 83.34	50	1112	23095	2.16	1	-

	83.34-93.33	85	1335	27824	3.05	1.32	0.93-1.88

	93.34-103.33	85	918	18885	4.5	1.75	1.23-2.50

	≥ 103.34	171	873	16484	10.37	3.49	2.51-4.86

Cigarette dosage (cigarettes/day)	Non-smoker	85	1126	23280	3.65	1	-

	1-9	89	1054	21334	4.17	1.37	1.02-1.85

	10-19	153	1691	33991	4.5	1.47	1.12-1.92

	≥ 20	64	367	7683	8.33	2.43	1.75-3.38

TC (mg/dl)	< 180	128	1879	38362	3.34	1	-

	180-199.9	90	931	18968	4.74	1.14	0.87-1.50

	200-219.9	77	682	13889	5.54	1.25	0.94-1.66

	≥ 220	96	746	15069	6.37	1.42	1.08-1.85

BMI (kg/m^2^)	-	391	4238			1.06	1.02-1.09

Adjusted for age, TC, BMI, SBP, and daily cigarette dosage.

Table 3 shows the results about BMI according to the WHO classification and the Chinese classification for underweight, normal weight, overweight and obesity. The average BMI in this study sample was 23.2 kg/m^2^; 35.0% of participants were overweight (BMI ≥ 24 kg/m^2^), and 5.2% were obesity (BMI ≥ 28 kg/m^2^). The HR for overweight and obesity was 1.24 (95% CI, 1.01-1.52) and 1.37 (95% CI, 0.96-1.97), respectively. When treated as a continuous variable, the HR for CVD was 1.16(95%CI, 0.99-1.37) for 5 points rise of BMI (Table 4).

**Table 3:** Two types of classification of adult underweight, overweight and obesity

	BMI (kg/m^2^)	CVD events	Number	Person years	Incidence (per 1000 person years)	HR	95%CI	*P *value
The WHO classification †								

Underweight	< 18.5	5	99	1999	2.5	0.7	0.29-1.69	0.43

Normal range	18.5-24.9	254	3151	64748	3.92	1	-	

Overweight	≥ 25.0	132	988	19541	6.76	1.25	1.00-1.55	< 0.05

Pre-obesity	25.0-29.9	120	929	18450	6.5	1.22	0.97-1.52	0.09

Obesity	≥ 30.0	12	59	1091	11	1.72	0.96-3.08	0.07

The Chinese Classification ‡								

Underweight	< 18.5	5	99	1999	2.5	0.72	0.29-1.74	0.46

Normal range	18.5-23.9	204	2655	54726	3.73	1	-	

Overweight	≥ 24.0	182	1484	56725	3.21	1.24	1.01-1.52	< 0.05

Pre-obesity	24.0-27.9	145	1265	25398	5.71	1.21	0.97-1.50	0.09

Obesity	≥ 28.0	37	219	4165	8.88	1.37	0.96-1.97	0.09

Adjusted for age, TC, BMI, SBP, and daily cigarette dosage.

† According to World Health Organization' web http://apps.who.int/bmi/index.jsp?introPage=intro_3.html

‡ According to Chinese overweight and obesity prevention guideline

**Table 4:** Multivariable adjusted hazard ratios (95% CI) for CVD from selected studies for men

Characteristics	Chinese steelworkers cohort		derivation cohort of QRISK2 model	
	HR	95% CI	HR	95% CI

Age (5 years increase)	1.29	1.18-1.40	1.59	1.58-1.60

SBP (20 mmHg increase)	1.63	1.48-1.80	1.19	1.17-1.20

Current smoker	1.54	1.21-1.97	1.65	1.60-1.70

TC	1.13 †	1.04-1.23	1.19 ‡	1.18-1.20

BMI (5 kg/m^2 ^increase)	1.16	0.99-1.37	1.09	1.07-1.11

Age* SBP interaction	0.993	0.920-1.070	0.964	0.960-0.969

Age* smoking interaction	1.211	1.010-1.451	0.932	0.922-0.942

Age* BMI interaction	0.955	0.929-0.981	0.985	0.979-0.991

Adjusted for age, TC, BMI, SBP, and Current smoker. † TC for 20 mg/dL increase, ‡ For Cholesterol/HDL ratio

Table 4 also shows the comparison between the results of Chinese steelworkers cohort and the results of males from the derivation cohort of QRISK2 model. The most difference was that the HR of CVD associated with every 20 mmHg rise in systolic blood pressure (SBP) in our study was much higher than result of QRISK2 model(1.63 vs. 1.19).

The PAR of hypertension, smoking, overweight (BMI ≥ 24 kg/m^2^) and hypercholesterolemia (> 220 mg/dl or 5.7 mmol/l) was 29.2%, 29.4%, 7.7% and 4.8%, respectively for CVD when using the risk factors prevalence of our study. While using the prevalence rates of risk factors in urban men in the National Health and Nutrition Survey 2002, the PAR for hypertension, smoking, overweight and hypercholesterolemia was 20.8%, 22.7%, 6.9% and 1.1%, respectively.

## Discussion

Since the Framingham heart study examined major risks of CVD in white American[15], a few studies at the population level in exploring different patterns of CVD risks between Chinese population and Caucasians have been reported[7,8,16,17], and risk scores for local population other than the Framingham score have subsequently been published[18,19]. Liu, et al [17] recalibrated the Framingham functions by using the mean values of risk factors and mean CHD incidence rate from Chinese Multi-Provincial Cohort Study (CMCS), given a much lower CHD incidence rate in China compared with that in Framingham, the United States. Our previous reports [7,8] also indicated that stroke was more prevalent than CHD in China by a cohort with an average 13.5 years follow-up. Furthermore, application of recalibrated Framingham models for ischemic cardiovascular disease (including ischemic stroke and coronary events) significantly overestimated the CHD risk in another Chinese cardiovascular epidemiology cohort [16]. It is controversial whether a recalibrated Western risk function can be useful in China. Except that risk prediction using CMCS and PRC-USA risk coefficients differed from Framingham, there were differences between the two Chinese cohort studies as well. In fact, the magnitude of risk coefficients predicting CVD in Chinese adults are not established. Therefore this study adds important information regarding the epidemiology of CVD and its risk factors in China. Furthermore, to our knowledge, this cohort followed up for an average of 20.84 years is the longest Chinese cohort specified for CVD events.

Prevalences of hypertension, hypercholesterolemia, and obesity in our cohort were lower than that in Caucasian cohorts, such as the MONICA cohort in Augsburg, Germany, but the prevalence of hypertension was still higher than that in China National Nutrition and Health Survey 2002 (32% vs. 20%). Smoking was much more prevalent in our cohort. The smoking rate in our cohort (73.4%) was similar with that of urban men from the PRC-USA cooperative study (71%-78%) [20], but higher than that for male adults who were 40 years of age or older in China National Hypertension Survey 1991(59.1%) [21].

In this study, we focused on magnitude of classical risk factors for CVD. We also compared our results with the HRs of QRISK2 model in derivation cohort (Table 4). QRISK2 was a prospective cohort study conducted in a large UK primary care population (2.3 million people aged 35-74 with 140 000 cardiovascular events) [11]. Although the QRISK2 model was derived mostly from European, there were still 22,013 south Asian and 19,792 from Chinese or other Asian or other ethnic groups among the overall population. Meanwhile, the characteristics of Chinese-English in derivation cohort of QRISK2 were similar with our baseline characteristics, for example, SBP (125.0 vs. 122.9 mmHg), BMI (23.4 vs. 23.2 kg/m^2^) and age at entry (49.0 vs. 44.9). Therefore, we compared the magnitude of classical risk factors in these two studies.

### Blood Pressure

The HR (1.30, 95% CI: 1.23-1.37) of CVD events for 10 mmHg rise in SBP in our cohort was higher than that (1.19, 95% CI: 1.17-1.20) in Caucasian study [11]. Our study indicated raised blood pressure is the leading preventable risk factor for CVD in the Chinese general population. There was a continuous graded increase in risk of CVD as blood pressure increased, with no evidence of a threshold value. This is consistent with the findings in the studies of Chinese and Asian population [17,22,23]. In QRISK2, there was an interaction between SBP and age on CVD. However, the interaction between SBP and age on CVD did not reach statistical significance in our study.

We also reported HRs of DBP, MAP and PP for every 10 mmHg rise in the prediction of CVD. These four BP indices represent different clinical meanings. The Physicians' Health Study suggested that SBP, DBP, and MAP strongly predict CVD among men younger than 60 years[24], whereas most studies showed that PP is a more useful and independent predictor for the risk of CVD in older people[25-28].

### Smoking

Cigarette smoking is another important risk factor of CVD for men in China because of its high prevalence. A nationally representative sample of Chinese adult cohort has reported RR of CVD for male cigarette smoker [29]. Though adjusted HRs for smoking in men of the Framingham Heart Study and the QRISK2 model were higher than that in our cohort (1.92 and 1.65 vs. 1.54) [11,30], our result was similar to that of Chinese national hypertension surveys [29].

The dose-response relationship between tobacco smoking and HR of CVD was observed in our study (Table 2). The effect of cigarette smoking on cardiovascular health is evident even at the lowest levels of exposure in Western study [31]. Similarly, the HR was statistically significant in the lowest cigarette dosage group (1-9 cigarettes/day) in our cohort. Moreover, an interaction between age and smoking on CVD was identified in our study (Table 4), which was different from the result of QRISK2 model. This additive interaction indicated that Chinese male smokers would have extra risk of CVD with the increase of age, and also implicated that smoking cessation may provide more benefits for CVD prevention in China.

### Total Cholesterol

A report indicated that 23.8% and 9.0% of the Chinese adult population have a total cholesterol level between 200 mg/dL and 239 mg/dL and ≥ 240 mg/dL, respectively [32], which was similar with our results (24.7% and 9.0%). The multivariable adjusted HR of TC in our study was similar to that of Caucasians. However the baseline level of TC in our study was much lower than that in the Framingham study and QRISK study, the role of hypercholesterolemia for CVD in Chinese was not as high as in Caucasians.

With decreasing cholesterol levels, previous study reported a trend towards an increase in risk of hemorrhagic stroke [23]. Although 21.8% of the participants with the serum TC level below 160 mg/dl, no such trend was detected in our current study (data not shown). Our result was consistent with the Heart Protection Study [33].

### Body Mass Index

Although overweight and obesity were not included in the general CVD risk prediction model of the Framingham Heart Study [30], the impact of body mass index on the risk of CVD incidence was proven by many studies. European guidelines on cardiovascular disease prevention suggested that avoiding overweight or reducing existing overweight should be important in patients with established CVD as well as in high risk people [34]. BMI was used as a predictive parameter for CVD in QRISK and QRISK2 models. The magnitude of BMI for CVD in our cohort was even greater than that in QRISK2. In QRISK2, there was a negative interaction between BMI and age on risk of CVD (0.985, 95% CI: 0.979-0.991), similar finding was found in our population (Table 4).

Participants of our study had a lower average BMI than those of most Caucasians studies [4,11,19,35]. For example, the average BMI in our study was 3.1 kg/m^2 ^lower than that of males in Framingham Heart Study. While there were positive associations between overweight and CVD either according to WHO definition or according to Chinese definition. However, using WHO classification might underestimate the number of obesity in Chinese population. In fact, there were only 59 participants (1.4%) with BMI ≥ 30 kg/m^2 ^in our cohort. Using 24 kg/m^2 ^and 28 kg/m^2 ^as the cut points for overweight and obesity in Chinese adults was recommended by the Chinese overweight and obesity prevention guideline, and was applied in many previous studies [36-38].

Overall, the role of overweight for CVD was confirmed in our study, and body weight controlling would reduce the incidence of CVD in Chinese males.

Generally speaking, using prevalence rates of these risk factors 20 year ago to estimate PARs of risk factors might be influenced by the changing of prevalence rates in this population. However, the PAR estimates using the risk factor levels of urban men in the National Health and Nutrition Survey 2002 were lower than expected. Compared with our results, the most difference was the PAR for hypercholesterolemia. Because the prevalence rate of hypercholesterolemia was only 3.7% in that national survey, it would obviously underestimate individual's number in our study (17.6%) if using the prevalence of the survey 2002. Other risk factors prevalence was also higher than that in national survey 2002 (hypertension, 31.7% vs. 20.2%, smoking 73.4% vs. 51.7%, overweight 35.0% vs. 31.1%). Considering the higher living standard in Beijing, it is reasonable that the prevalence of CVD risk factors in our cohort advanced the average levels in China. Therefore, the PARs derived from our study have much more practical significance.

## Limitations

Some limitations of our study should be noted. Although prospective and with a long follow-up time, the Beijing Iron and Steel cohort study is workplace based and therefore not necessarily representative of the general population. After the 1978 economic reforms, China has experienced very rapid economic growth. The consequent improvement in living conditions, nutrition, and health care in the past several decades would affect risk factor levels and result in epidemiologic transition of diseases pattern. Though we have used the risk factor levels of urban men in the National Health and Nutrition Survey 2002 to calculate the PARs, it should be caution when applied those results into the general population. In addition, our cohort was restricted with very limited number of diabetes, and total cholesterol alone was measured because high density lipoprotein (HDL) cholesterol test was not widely used in China during the 1980s. Thus, the impacts of diabetes and HDL cholesterol on cardiovascular disease risk could not be assessed.

## Conclusions

Our prospective data demonstrates that hypertension, cigarette smoking, overweight, hypercholesterolemia, are major risk factors for CVD. Hypertension and Cigarette smoking are the leading predictive parameter for CVD in Chinese male adults, and the association between blood pressure and CVD in Chinese is stronger than in Caucasians. To reduce incidence of CVD in Chinese males, continued strengthening of programs for prevention and intervention on these risks is needed.

## Competing interests

The authors declare that they have no competing interests.

## Authors' contributions

JJ analysed the data and drafted the manuscript. EP, JC, JL, JC, DS, DG, XW, XD and JH conducted the investigation and participated in discussing. JH supervised the project and revised the manuscript. All authors read and approved the final manuscript.

## Pre-publication history

The pre-publication history for this paper can be accessed here:

http://www.biomedcentral.com/1471-2458/11/497/prepub
